# Genome Analysis of Triploid Hybrid *Leishmania* Parasite from the Neotropics

**DOI:** 10.3201/eid2905.221456

**Published:** 2023-05

**Authors:** Frederik Van den Broeck, Senne Heeren, Ilse Maes, Mandy Sanders, James A. Cotton, Elisa Cupolillo, Eugenia Alvarez, Lineth Garcia, Maureen Tasia, Alice Marneffe, Jean-Claude Dujardin, Gert Van der Auwera

**Affiliations:** Katholieke Universiteit Leuven, Leuven, Belgium (F. Van den Broeck, S. Heeren);; Institute of Tropical Medicine, Antwerp, Belgium (F. Van den Broeck, S. Heeren, I. Maes, J.-C. Dujardin, G. Van der Auwera);; Wellcome Sanger Institute, Hinxton, UK (M. Sanders, J.A. Cotton);; Instituto Oswaldo Cruz–Fiocruz, Rio de Janeiro, Brazil (E. Cupolillo);; Cayetano Heredia University, Lima, Peru (E. Alvarez);; Universidad Mayor de San Simon, Cochabamba, Bolivia (L. Garcia);; Centre Hospitalier Universitaire St. Pierre, Brussels, Belgium (M. Tasia, A. Marneffe)

**Keywords:** leishmaniasis, genomics, genetics, interspecific hybridization, *Leishmania*, cutaneous parasites, Costa Rica, Panama, Belgium

## Abstract

We discovered a hybrid *Leishmania* parasite in Costa Rica that is genetically similar to hybrids from Panama. Genome analyses demonstrated the hybrid is triploid and identified *L. braziliensis* and *L. guyanensis*–related strains as parents. Our findings highlight the existence of poorly sampled *Leishmania* (*Viannia*) variants infectious to humans.

*Leishmania* are intracellular protozoan parasites that cause the vectorborne disease leishmaniasis, which occurs in ≈88 countries (*1*). Human infection can result in 2 main forms of disease, cutaneous and visceral leishmaniasis, and different *Leishmania* species cause diverse clinical manifestations and sequelae (*1*). Correct species typing is thus required to clinically manage leishmaniasis (*2*).

In August 2020, a patient returning from Costa Rica sought care at the Hospital St. Pierre (Brussels, Belgium) with a single, well-demarcated, ulcerated erythematous plaque on the left flank indicative of cutaneous leishmaniasis. Molecular diagnosis confirmed the presence of *Leishmania* parasites on the basis of 18S ribosomal DNA (*3*). A 1,245-bp fragment of the multicopy heat-shock protein 70 gene (*hsp70*) was sequenced for species typing (*4*). This sequencing revealed an atypical sequence related to both the *L. guyanensis* and *L. braziliensis* species that showed sequence variation across copies at 10 positions, suggesting either a mixed infection or hybrid parasite. Despite the atypical nature of the infecting species, the patient had good therapeutic response after 5 intralesional injections with meglumine antimoniate (Glucantime, Sanofi, https://www.sanofi.com), leaving only a slightly hyperpigmented scar. The clinical sample was cultured in vitro (referred to as MHOM/CR/2020/StPierre) and subjected to HSP70 typing (*4*) and whole-genome sequencing (*5*).

Compared with results for the clinical sample, the consensus sequence of the *hsp70* locus in the cultured isolate revealed sequence variation in 1 extra site, bringing the total to 11 (Appendix 1). Of those sites, 10 were shared with 6 cutaneous leishmaniasis strains described from Panama (*6*). Comparison with all available *Leishmania hsp70* sequences from GenBank (Appendix 1) revealed 2 monophyletic groups as the possible origin of the different *hsp70* copies in the Costa Rica sequence: first, a subgroup of the *L. guyanensis* species complex found in Ecuador, Panama, and Colombia; and second, a subgroup of the *L. braziliensis* species complex described from Panama, Guatemala, and Brazil. Even though such analysis is biased by available sequences and the use of a single chromosomal locus, the geography of the hypothetical parents is compatible with Costa Rica. To further investigate the nature of the Costa Rica isolate, we resorted to genome analysis.

We identified 125,632 single-nucleotide polymorphisms (SNPs) within the sample from Costa Rica after mapping genomic sequences against the *L. braziliensis* M2904 reference genome (*5*) (Appendix 2). This total included 21,168 homozygous SNPs (both haplotypes were different from M2904) and 104,464 heterozygous SNPs (one haplotype was similar to M2904 and the other different). Chromosomes 1 and 11, the first 140 kb of chromosome 20, and the last 60 kb of chromosome 27 were highly homozygous, almost completely lacking in heterozygous variants, whereas most variants in the rest of the genome were heterozygous (Appendix 2). This observation of a largely heterozygous genome that is interrupted by homozygous stretches strongly suggests that the isolate is a hybrid parasite, rather than the result of a mixed infection (*7*).

We investigated chromosome copy numbers by using the distribution of allelic read depth frequencies at heterozygous sites (*7*), which should be centered around 0.5 in diploid organisms (both alleles represented equally). However, we discovered a bimodal distribution with modes 0.33 and 0.67 (Appendix 2), suggesting that the hybrid is triploid (*7*).

We analyzed the genomic ancestry of the hybrid compared with 40 genomes of 7 *Leishmania* (*Viannia*) species (Appendix 2) using phylogenies based on genomic regions that were homozygous in the hybrid (where both haplotypes originate from the same parental species). In the chromosome 20 phylogeny, the Costa Rica hybrid clusters with *L. braziliensis* 1 strain Lb8102 from Colombia (Figure; Appendix 2 Figure 8), which could be close to one of the parental strains. The ancestry of the other parental genome remains unclear, because in the chromosome 1 phylogeny it clusters with *L. panamensis* strains from Colombia and Panama (Figure), even though it is also tightly linked with a cluster of *L. guyanensis* strains from Venezuela, Brazil, and French Guiana in the mitochondrial maxicircle phylogeny (Appendix 2 Figures 3, 9). We could not resolve the geographic origin of the 2 parents in greater detail because of the lack of available *Leishmania* (*Viannia*) genomes.

**Figure:**
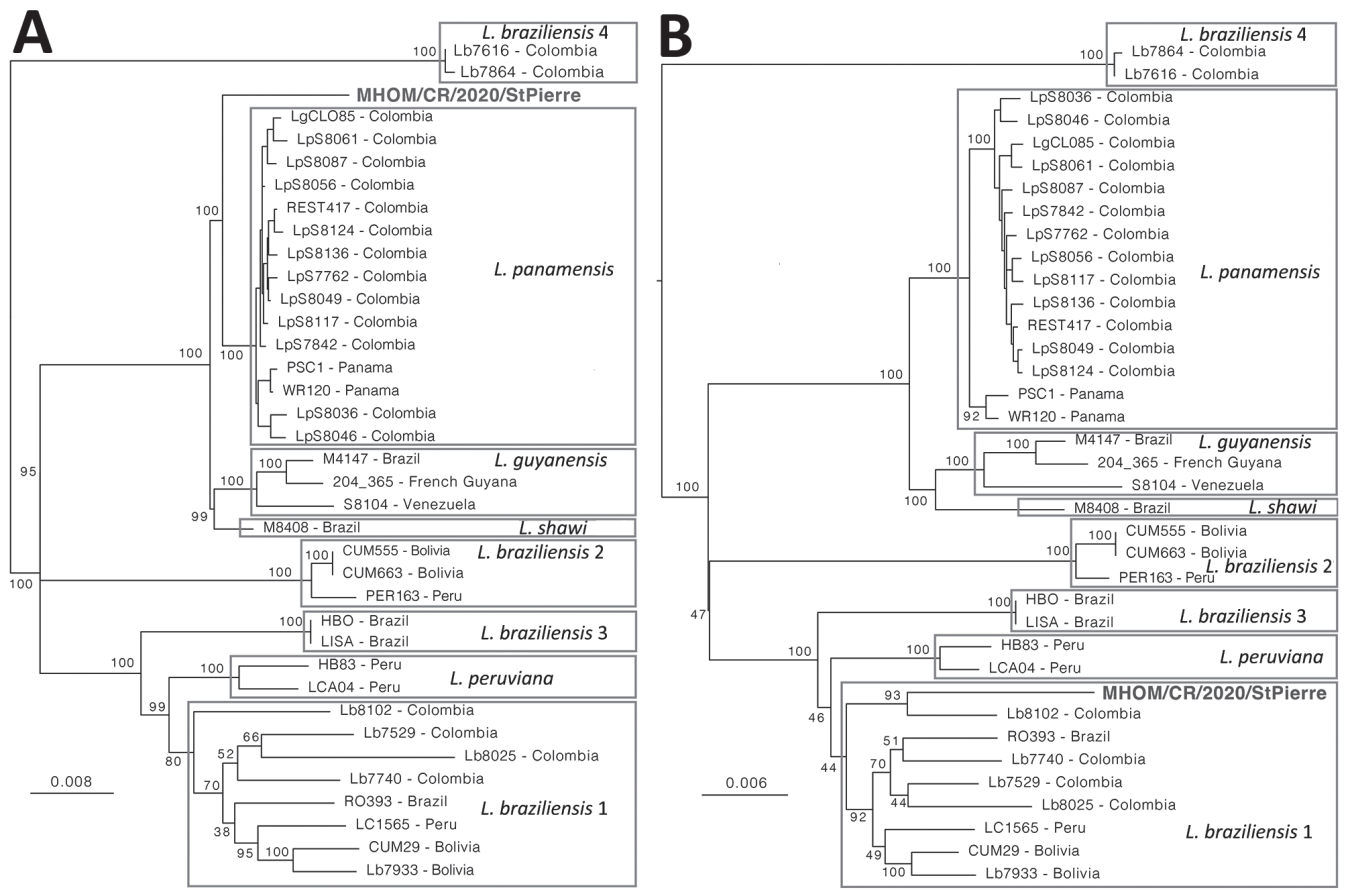
Midpoint rooted maximum-likelihood phylogenetic trees based on single-nucleotide polymorphisms called in chromosome 1 and the first 140kb of the telomeric region of chromosome 20 of a hybrid *Leishmania* parasite from Costa Rica. For each strain, sequences were composed based on concatenated single-nucleotide polymorphisms that were each coded by 2 base pairs, after which invariant sites were removed, resulting in 2,382 bp sequences for chromosome 1 and 3,015 bp sequences for chromosome 20. Consensus phylogenetic trees were generated from 1,000 bootstrap trees using IQ-TREE (http://www.iqtree.org) with 37 taxa (excluding *L. naiffi* and *L. lainsoni* strains) under the  transversion with empirical base frequencies, ascertainment bias correction, and discrete gamma with 4 rate categories substitution model, which was the best-fit model revealed by ModelFinder as implemented in IQTREE. Branch support values are presented near each node following 1,000 bootstrap replicates; bootstrap values within the clade containing *L. panamensis* strains were omitted for clarity reasons. Scale bar indicates number of substitutions per site. Appendix 2) includes a description of the *L. braziliensis* 1–4 lineages.

Our study provides a detailed genomic description of a hybrid between the *L. braziliensis* and the *L. guyanensis* species complexes. The first report of such hybrids in Central America dates back to the early 1990s, concerning putative *L. braziliensis*–*L. panamensis* hybrids from the north of Nicaragua (*8*). Those hybrids were reported again in 2021 in Panama (*6*). Further, parasites with signatures from both *L. braziliensis* and *L. guyanensis* relatives have been described in South America, more specifically from Ecuador, Peru, Brazil, and Venezuela (*9*,*10*). Together with our report from Costa Rica, these reports point to a widespread circulation in the Neotropical region of recombinant strains, the epidemiology and clinical significance of which remain elusive.

Appendix 1 Additional *hsp70* data about emergence of hybrid *Leishmania* parasite from the Neotropics.

Appendix 2 Additional whole genome information about genome analysis of triploid hybrid *Leishmania* parasite from the Neotropics.
