# Supplementary material for: Genome Analysis of Triploid Hybrid Leishmania Parasite from the Neotropics
Source: Emerg Infect Dis. 2023 May;29(5):1076–8. doi: 10.3201/eid2905.221456 (PMC10124652; doi:10.3201/eid2905.221456)
Supplement: Appendix 1 — Additional hsp70 data about emergence of hybrid Leishmania parasite from the Neotropics. [file 22-1456-Techapp-s1.pdf]

Article DOI: https://doi.org/10.3201/eid2905.221456

EID cannot ensure accessibility for supplementary materials supplied by authors. Readers who have difficulty accessing supplementary content should contact the authors for assistance.

Genome Analysis of Triploid Hybrid *Leishmania* Parasite from Neotropics

Appendix 1

The following pages provide further details from the *hsp70* analysis of a hybrid *L. guyanensis* complex–*L. braziliensis* complex strain isolated in 2020 from Costa Rica.

Heat-shock protein 70 gene analysis of isolate MHOM/CR/2020/StPierre

- Returning cutaneous leishmaniasis patient from Costa Rica.
- Partial heat-shock protein 70 gene (*hsp70*) sequence (1245 bp) of the isolated parasite contained **11 positions** with more than 1 nucleotide present, resulting from sequence variation between the multiple copies of the tandemly repeated gene (GenBank accession OQ200658):

```
GGCCTRGAGG TGCTGCGCAT CATCAACGAG CCAACAGCGG CGGCCATCGC GTACGGCCTG GACAAGGGCG ACGACGGCAA GGAGCGCAAC GTGCTCATCT  100
TCGACCTTGG CGGCGGCACG TTCGATGTGA CGCTGCTGAC GATCGACGGC GGCATCTTCG AGGTGAAGGC GACGAACGGT GACACGCACC TTGGCGGCGA  200
GGACTTTGAC AACCGCCTCG TCACGTTCTT CASCGAGGAG TTCAAGCGCA AGAACAAGGG TAARGACCTT TCGTCGAGCC ACCGCGCGCT GCGCCGCCTG  300
CGCACGGCGT GCGAGCGCGC GAAGCGCACG CTGTCGTCCG CGACGCAGGC GACGATCGAG ATCGACGCGC TGTTCGACAA CGTCGACTTC CAGGCCAACA  400
TCACGCGCGC GCGCTTCGAG GAGCTGTGCG GCGACCTGTT CCGCAGCACA ATGCAGCCGG TGGAGCGCGT GCTGCAGGAC GCGAAGATGG ACAAGCGCTC  500
CGTGCACGAC GTGGTGCTGG TGGGCGGGTC GACGCGCATC CCGAAGGTGC AGTCCCTCGT GTCGGACTTC TTCGGCGGCA AGGAGCTGAA CAAGAGCATC  600
AACCCCGACG AGGCTGTCGC GTACGGCGCT GCGGTGCAGG CGTTCATCCT GACGGGCGGC AAGAGCAAGC AGACRGAGGG CCTGCTGCTG CTGGATGTGA  700
CGCCGCTGAC CCTGGGCATT GAGACGGCCG GCGGCGTGAT GACGGCGCTG ATCAAGCGCA ACACGACGAT CCCGACCAAG AAGAGCCAGA TCTTCTCGAC  800
GTACGCGGAC AACCAGCCCG GCGTGCACAT CCAGGTCTTC GAGGGCGAGC GCGCGATGAC GAAGGACTGC CACCTGCTGG GCACGTTCGA CTTGTCCGGC  900
ATCCCGCCAG CGCCGCGCGG CGTGCCGCAG ATCGAGGTGA CGTTCGACCT GGACGCGAAC GGCATCCTGA ACGTGTCCGC GGAGGAGAAG GGCACCGGCA 1000
AGCGCAACCA TATCACCATC ACCAACGACA AGGGCCGACT GAGCAAGGAC GAGATCGAGC GCATGGTGAA CGATGCGKCG AAGTACGAGC AGGCCGACAA 1100
GRTGCAGCGC GAGCGCGTGG AGGCGAAGAA CGGYCTGGAG AACTACGCGT ACTCGATGAA GAACACGRTC KCCGACACGA RCGTGTCCGG CAAGCTGGAG 1200
GAGAGCGACA GGACCGCGCT GAACTCGGCG ATCGASGCGG CGCTG                                                          1245
```

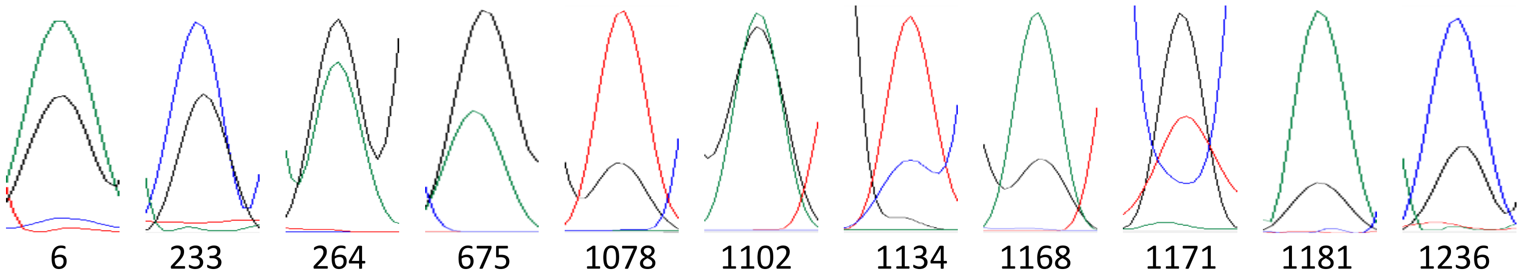

Example of sequence electropherogram for each ambiguous position

- Sequence comparison excluding the ambiguous sites from the alignment links the isolate with the *L. guyanensis* and *L. braziliensis* species complexes.

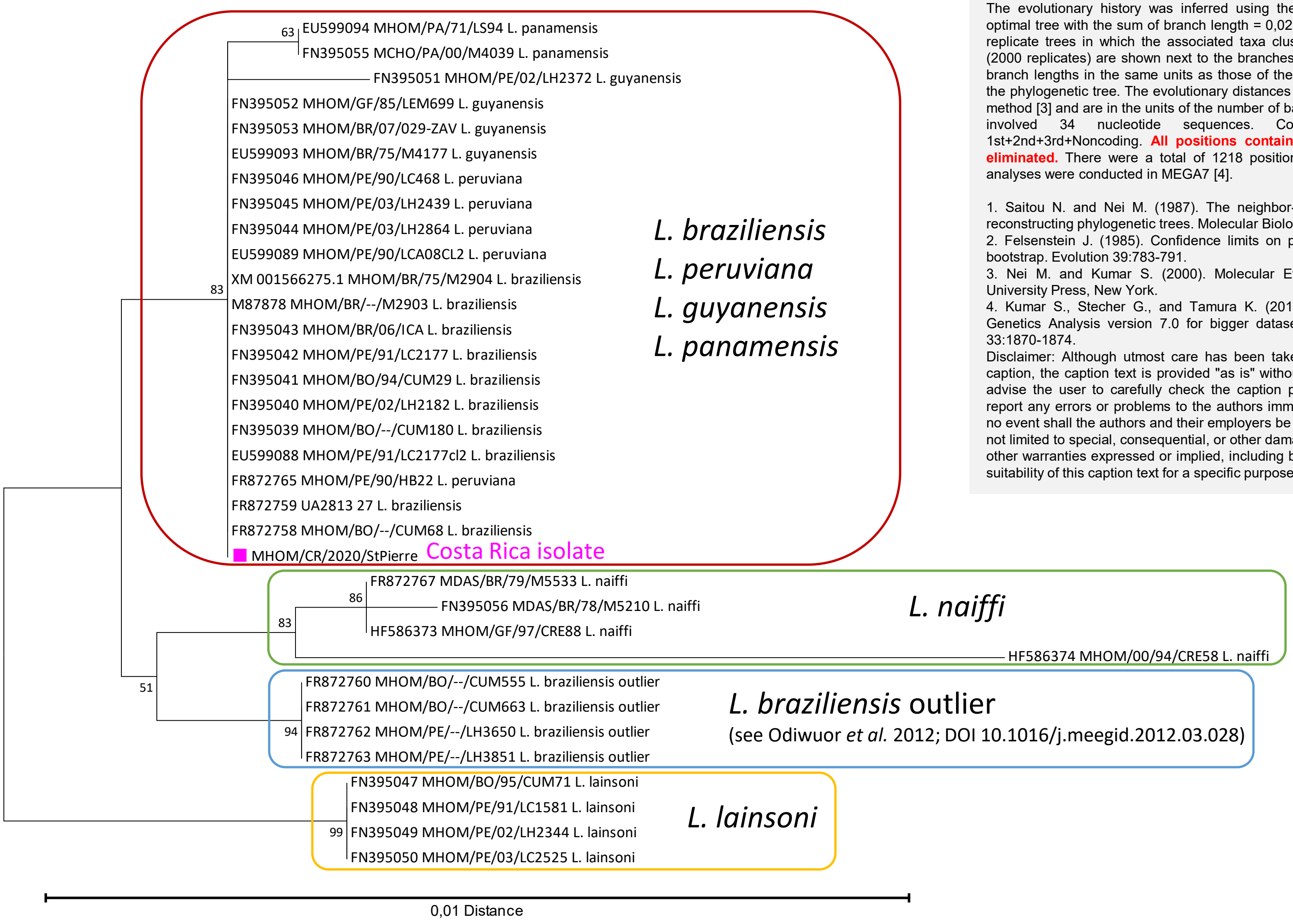

Evolutionary relationships of taxa

The evolutionary history was inferred using the Neighbor-Joining method [1]. The optimal tree with the sum of branch length = 0,02257800 is shown. The percentage of replicate trees in which the associated taxa clustered together in the bootstrap test (2000 replicates) are shown next to the branches [2]. The tree is drawn to scale, with branch lengths in the same units as those of the evolutionary distances used to infer the phylogenetic tree. The evolutionary distances were computed using the p-distance method [3] and are in the units of the number of base differences per site. The analysis involved 34 nucleotide sequences. Codon positions included were 1st+2nd+3rd+Noncoding. **All positions containing gaps and missing data were eliminated.** There were a total of 1218 positions in the final dataset. Evolutionary analyses were conducted in MEGA7 [4].

1. Saitou N. and Nei M. (1987). The neighbor-joining method: A new method for reconstructing phylogenetic trees. Molecular Biology and Evolution 4:406-425.
2. Felsenstein J. (1985). Confidence limits on phylogenies: An approach using the bootstrap. Evolution 39:783-791.
3. Nei M. and Kumar S. (2000). Molecular Evolution and Phylogenetics. Oxford University Press, New York.
4. Kumar S., Stecher G., and Tamura K. (2016). MEGA7: Molecular Evolutionary Genetics Analysis version 7.0 for bigger datasets.Molecular Biology and Evolution 33:1870-1874.
Disclaimer: Although utmost care has been taken to ensure the correctness of the caption, the caption text is provided "as is" without any warranty of any kind. Authors advise the user to carefully check the caption prior to its use for any purpose and report any errors or problems to the authors immediately (www.megasoftware.net). In no event shall the authors and their employers be liable for any damages, including but not limited to special, consequential, or other damages. Authors specifically disclaim all other warranties expressed or implied, including but not limited to the determination of suitability of this caption text for a specific purpose, use, or application.

- This indicates either a hybrid strain or mixed infection.

- To identify the parental sequences of the hybrid or the mix, all *hsp70* sequences spanning the full 1245 bp fragment from GenBank were analyzed (January 31, 2022), supplemented with patient sequences from the Institute of Tropical Medicine (Antwerp, Belgium).
- In the 11 ambiguous positions, several nucleotides were found only in a subset of *L. guyanensis* from Ecuador (further called **parent 1b**), and equally so in an unidentified group of sequences from Panama (published in Miranda *et al.* 2021) (**parent 1a**).
- If these sequences are indeed a parent, all other nucleotides in the ambiguous positions need be accounted for by the second parent, leaving only a subgroup of *L. braziliensis* as the second possible parent (called further **parent 2**).

Table: comparison of MHOM/CR/2020/StPierre with putative parental sequences at the 11 ambiguous sites. Identification of the parental sequences: please refer to legend of figure on next page. Number of identical sequences in the analysis is in brackets following sequence descriptions.

Position in partial 1245 bp fragment of *hsp70* →	6	233	264	675	1078	1102	1134	1168	1171	1181	1236
Position in HSP70 CDS of whole genome →	504	731	762	1173	1576	1600	1632	1666	1669	1679	1734
MHOM/CR/2020/StPierre	R	S	R	R	K	R	Y	R	K	R	S
Parent1b 1 L guyanensis complex Panama(1)	A	C	G	A	G	G	T	A	G	G	G
Parent1b 2 L guyanensis complex Panama(22)	A	C	G	A	G	G	T	A	G	G	G
Parent1b 3 L guyanensis complex Panama(1)	A	C	G	A	G	G	T	A	G	R	G
Parent1b 4 L guyanensis complex Panama(13)	A	C	G	A	G	G	T	A	G	G	G
Parent1b 5 L guyanensis complex Colombia(1)	A	C	G	R	K	R	T	A	G	R	S
Parent1a 6 L guyanensis complex Ecuador(1)	A	C	G	A	G	G	T	A	R	R	G
Parent1a 7 L guyanensis complex Ecuador(1)	A	C	G	A	G	G	T	A	G	R	G
Parent1a 8 L guyanensis complex Ecuador(2)	A	C	G	A	G	G	T	A	R	R	G
Parent1a 9 L guyanensis complex Ecuador(1)	A	C	G	A	G	G	T	A	G	G	G
Parent1a 10 L guyanensis complex Ecuador(2)	A	C	G	A	G	G	T	A	G	R	G
Parent2 11 L braziliensis Guatemala(3)	G	G	A	G	T	A	C	G	T	A	C
Parent2 12 L braziliensis Panama(1)	G	G	A	G	T	A	C	G	T	A	C
Parent2 13 L braziliensis Panama(1)	G	G	A	G	T	A	C	G	T	A	C
Parent2 14 L braziliensis(1)	G	G	A	G	T	A	C	G	T	A	C
Parent2 15 L braziliensis Brazil(7) Panama(1)	G	G	A	G	T	A	C	G	T	A	C

- Not only could the hypothetical parental lines account for all 11 of the ambiguous positions, but conversely, every single one of the 1245 positions in the mix/hybrid showed the nucleotide(s) present in both parents. In other words: all sequence variation in the parents was accounted for in the hybrid/mix sequence.
- Phylogenetic analysis of these hypothetical parental sequences gave the following results:

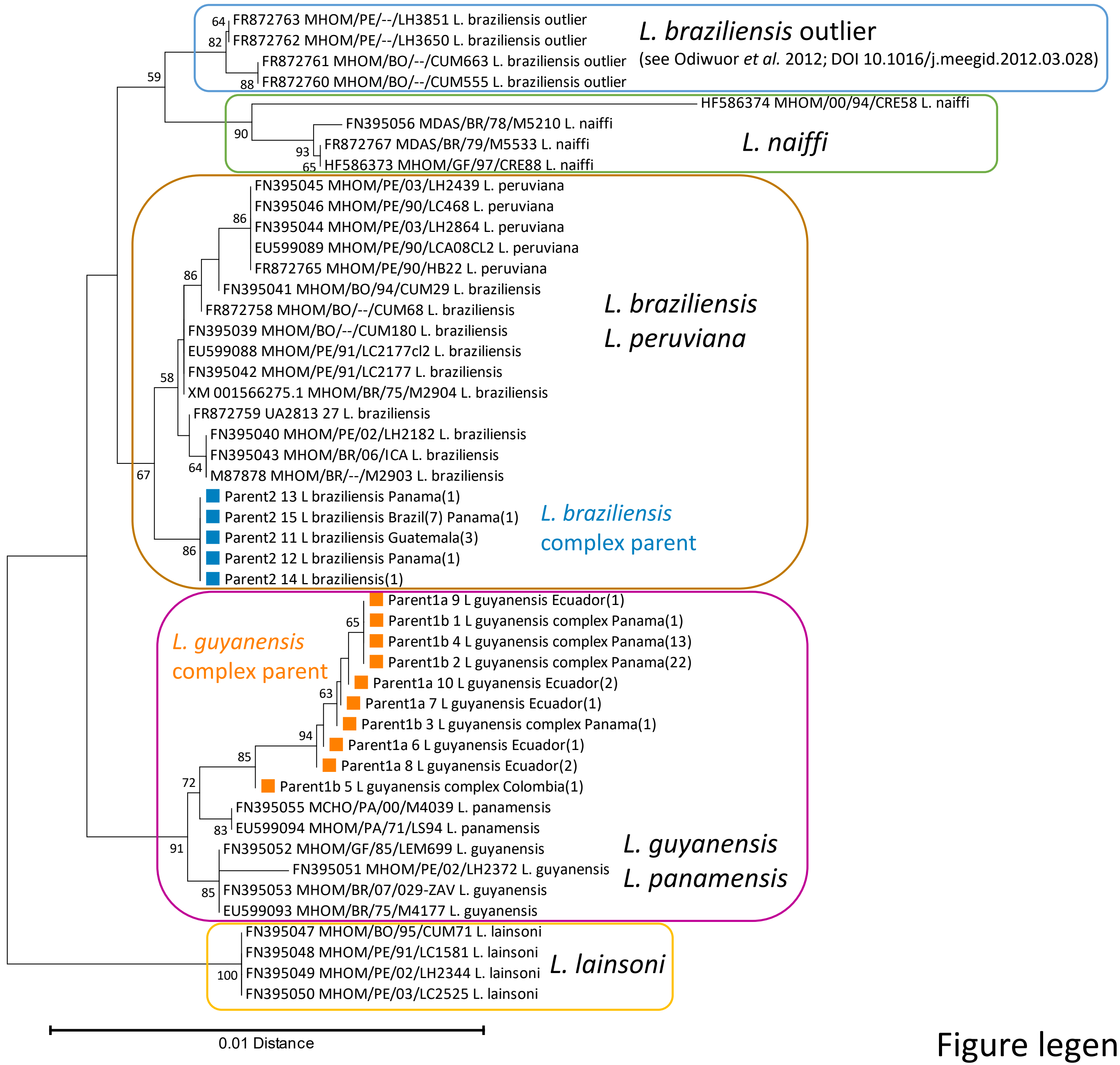

Figure legend: next page

Only 15 unique parental sequences were retained, each of these is identified as follows:

Parent (1a/1b/2) – unique sequence number (1-15) – species – country of origin (number of identical sequences represented in brackets).

Dendrogram method

The evolutionary history was inferred using the Neighbor-Joining method [1]. The optimal tree with the sum of branch length = 0.04003125 is shown. The percentage of replicate trees in which the associated taxa clustered together in the bootstrap test (2000 replicates) are shown next to the branches when exceeding 50% [2]. The tree is drawn to scale, with branch lengths in the same units as those of the evolutionary distances used to infer the phylogenetic tree. The evolutionary distances were computed using the p-distance method [3] and are in the units of the number of base differences per site. The analysis involved 48 nucleotide sequences. Codon positions included were 1st+2nd+3rd+Noncoding. **All ambiguous positions were removed for each sequence pair.** There were a total of 1245 positions in the final dataset. Evolutionary analyses were conducted in MEGA7 [4].

1. Saitou N. and Nei M. (1987). The neighbor-joining method: A new method for reconstructing phylogenetic trees. Molecular Biology and Evolution 4:406-425.
2. Felsenstein J. (1985). Confidence limits on phylogenies: An approach using the bootstrap. Evolution 39:783-791.
3. Nei M. and Kumar S. (2000). Molecular Evolution and Phylogenetics. Oxford University Press, New York.
4. Kumar S., Stecher G., and Tamura K. (2016). MEGA7: Molecular Evolutionary Genetics Analysis version 7.0 for bigger datasets.Molecular Biology and Evolution 33:1870-1874.
Disclaimer: Although utmost care has been taken to ensure the correctness of the caption, the caption text is provided "as is" without any warranty of any kind. Authors advise the user to carefully check the caption prior to its use for any purpose and report any errors or problems to the authors immediately (www.megasoftware.net). In no event shall the authors and their employers be liable for any damages, including but not limited to special, consequential, or other damages. Authors specifically disclaim all other warranties expressed or implied, including but not limited to the determination of suitability of this caption text for a specific purpose, use, or application.

The following entries are represented by sequences 1-15:

(if from GenBank, description starts with accession number)

1_L_guyanensis_complex_Panama(1)
KX574009.1:1-1245_Leishmania_sp._isolate_PA_462_heat_shock_protein_70_(hsp70)_gene_partial_cds
2_L_guyanensis_complex_Panama(22)
KX573944.1:1-1245_Leishmania_sp._isolate_PA_026_heat_shock_protein_70_(hsp70)_gene_partial_cds
KX573946.1:1-1245_Leishmania_sp._isolate_PA_066_heat_shock_protein_70_(hsp70)_gene_partial_cds
KX573947.1:1-1245_Leishmania_sp._isolate_PA_067_heat_shock_protein_70_(hsp70)_gene_partial_cds
KX573949.1:1-1245_Leishmania_sp._isolate_PA_073_heat_shock_protein_70_(hsp70)_gene_partial_cds
KX573952.1:1-1245_Leishmania_sp._isolate_PA_086_heat_shock_protein_70_(hsp70)_gene_partial_cds
KX573953.1:1-1245_Leishmania_sp._isolate_PA_090_heat_shock_protein_70_(hsp70)_gene_partial_cds
KX573957.1:1-1245_Leishmania_sp._isolate_PA_108_heat_shock_protein_70_(hsp70)_gene_partial_cds
KX573959.1:1-1245_Leishmania_sp._isolate_PA_113_heat_shock_protein_70_(hsp70)_gene_partial_cds
KX573961.1:1-1245_Leishmania_sp._isolate_PA_119_heat_shock_protein_70_(hsp70)_gene_partial_cds
KX573964.1:1-1245_Leishmania_sp._isolate_PA_131_heat_shock_protein_70_(hsp70)_gene_partial_cds
KX573965.1:1-1245_Leishmania_sp._isolate_PA_133_heat_shock_protein_70_(hsp70)_gene_partial_cds
KX573966.1:1-1245_Leishmania_sp._isolate_PA_137_heat_shock_protein_70_(hsp70)_gene_partial_cds
KX573967.1:1-1245_Leishmania_sp._isolate_PA_145_heat_shock_protein_70_(hsp70)_gene_partial_cds
KX573969.1:1-1245_Leishmania_sp._isolate_PA_162_heat_shock_protein_70_(hsp70)_gene_partial_cds
KX573970.1:1-1245_Leishmania_sp._isolate_PA_163_heat_shock_protein_70_(hsp70)_gene_partial_cds
KX573972.1:1-1245_Leishmania_sp._isolate_PA_169_heat_shock_protein_70_(hsp70)_gene_partial_cds
KX573973.1:1-1245_Leishmania_sp._isolate_PA_171_heat_shock_protein_70_(hsp70)_gene_partial_cds
KX573976.1:1-1245_Leishmania_sp._isolate_PA_197_l2_heat_shock_protein_70_(hsp70)_gene_partial_cds
KX573978.1:1-1245_Leishmania_sp._isolate_PA_224_l1_heat_shock_protein_70_(hsp70)_gene_partial_cds
KX573980.1:1-1245_Leishmania_sp._isolate_PA_233_heat_shock_protein_70_(hsp70)_gene_partial_cds
KX573982.1:1-1245_Leishmania_sp._isolate_PA_284_heat_shock_protein_70_(hsp70)_gene_partial_cds
KX573988.1:1-1245_Leishmania_sp._isolate_PA_357_heat_shock_protein_70_(hsp70)_gene_partial_cds
3_L_guyanensis_complex_Panama(1)
KX573990.1:1-1245_Leishmania_sp._isolate_PA_368_heat_shock_protein_70_(hsp70)_gene_partial_cds
4_L_guyanensis_complex_Panama(13)
KX573993.1:1-1245_Leishmania_sp._isolate_PA_397_heat_shock_protein_70_(hsp70)_gene_partial_cds
KX573994.1:1-1245_Leishmania_sp._isolate_PA_407_heat_shock_protein_70_(hsp70)_gene_partial_cds
KX573997.1:1-1245_Leishmania_sp._isolate_PA_415_heat_shock_protein_70_(hsp70)_gene_partial_cds
KX573998.1:1-1245_Leishmania_sp._isolate_PA_418_heat_shock_protein_70_(hsp70)_gene_partial_cds
KX573999.1:1-1245_Leishmania_sp._isolate_PA_425_heat_shock_protein_70_(hsp70)_gene_partial_cds
KX574002.1:1-1245_Leishmania_sp._isolate_PA_438_heat_shock_protein_70_(hsp70)_gene_partial_cds
KX574003.1:1-1245_Leishmania_sp._isolate_PA_439_heat_shock_protein_70_(hsp70)_gene_partial_cds
KX574004.1:1-1245_Leishmania_sp._isolate_PA_443_heat_shock_protein_70_(hsp70)_gene_partial_cds
KX574005.1:1-1245_Leishmania_sp._isolate_PA_444_heat_shock_protein_70_(hsp70)_gene_partial_cds
KX574006.1:1-1245_Leishmania_sp._isolate_PA_445_heat_shock_protein_70_(hsp70)_gene_partial_cds
KX574012.1:1-1245_Leishmania_sp._isolate_PA_469_heat_shock_protein_70_(hsp70)_gene_partial_cds
KX574014.1:1-1245_Leishmania_sp._isolate_PA_530_heat_shock_protein_70_(hsp70)_gene_partial_cds
KX574015.1:1-1245_Leishmania_sp._isolate_PA_539_heat_shock_protein_70_(hsp70)_gene_partial_cds
5_L_guyanensis_complex_Colombia(1)
LN907837.1:1-1245_Leishmania_sp._MHOM/CO/88/UA316_partial_hsp70_gene_for_heat-shock_protein_70_kDa_strain_MHOM/CO/88/UA316
6_L_guyanensis_complex_Ecuador(1)
MHOM/EC/2015/ITM15102466_organism_Leishmania_guyanensis
7_L_guyanensis_complex_Ecuador(1)
MHOM/EC/2015/ITM15121151_organism_Leishmania_guyanensis_complex
8_L_guyanensis_complex_Ecuador(2)
MHOM/EC/2015/ITM15121974_organism_Leishmania_guyanensis
MHOM/EC/2017/ITM17113319_organism_Leishmania_guyanensis
9_L_guyanensis_complex_Ecuador(1)
MHOM/EC/2018/ITM18093159_organism_Leishmania_guyanensis_complex
10_L_guyanensis_complex_Ecuador(2)
MN688569.1:1-1245_Leishmania_guyanensis_isolate_MHOM/EC/2016/BCN-885_heat-shock_protein_70_(hsp70)_gene_partial_cds
MT498900.1:1-1245_Leishmania_guyanensis_isolate_MHOM/EC/2016/BCN-885_heat_shock_protein_70_(hsp70)_gene_partial_cds
11_L_braziliensis_Guatemala(3)
MHOM/GT/2018/ITM18070595_organism_Leishmania_braziliensis
MN688566.1:1-1245_Leishmania_braziliensis_isolate_MHOM/GT/2005/BCN-717_heat-shock_protein_70_(hsp70)_gene_partial_cds
MT498877.1:1-1245_Leishmania_braziliensis_isolate_MHOM/GT/2005/BCN-717_heat_shock_protein_70_(hsp70)_gene_partial_cds
12_L_braziliensis_Panama(1)
KX573943.1:1-1245_Leishmania_sp._isolate_PA_024_heat_shock_protein_70_(hsp70)_gene_partial_cds
13_L_braziliensis_Panama(1)
KX573956.1:1-1245_Leishmania_sp._isolate_PA_107_heat_shock_protein_70_(hsp70)_gene_partial_cds
14_L_braziliensis(1)
MHOM/--/2017/ITM17092834_organism_Leishmania_braziliensis
15_L_braziliensis_Brazil(7)_Panama(1)
KX573991.1:1-1245_Leishmania_sp._isolate_PA_372_heat_shock_protein_70_(hsp70)_gene_partial_cds
MH745179.1:1-1245_Leishmania_braziliensis_strain_MHOM/BR/2001/BA788_heat_shock_protein_70_gene_partial_cds
MH745180.1:1-1245_Leishmania_braziliensis_strain_IWELL/BR/1981/M8401_heat_shock_protein_70_gene_partial_cds
MH745181.1:1-1245_Leishmania_braziliensis_strain_MHOM/BR/1996/M15991_heat_shock_protein_70_gene_partial_cds
MH745182.1:1-1245_Leishmania_braziliensis_strain_MHOM/BR/1995/RR80_heat_shock_protein_70_gene_partial_cds
MH745183.1:1-1245_Leishmania_braziliensis_strain_MHOM/BR/2008/426_heat_shock_protein_70_gene_partial_cds
MH745184.1:1-1245_Leishmania_braziliensis_strain_MHOM/BR/1995/RR051_heat_shock_protein_70_gene_partial_cds
MH745185.1:1-1245_Leishmania_braziliensis_strain_MHOM/BR/1975/M2903_heat_shock_protein_70_gene_partial_cds

- The putative parental haplotypes were confirmed partly by whole genome sequencing, using sequence reads covering several ambiguous sites.
- Interestingly, 7 sequences previously reported from Panama (3 of which published in [Miranda *et al.* 2021]) share up to 10 ambiguous positions with the Costa Rica sequence.
- The following figure shows (dash representing similarity with top sequence)
    - The ambiguous sites in the Costa Rica sequence.
    - The nucleotides in the 7 previously reported sequences from Panama (identified with GenBank entry).

Position in partial 1245 bp fragment of *hsp70* →	6	233	264	675	1078	1102	1134	1168	1171	1181	1236
Position in HSP70 CDS of whole genome →	504	731	762	1173	1576	1600	1632	1666	1669	1679	1734
MHOM/CR/2020/StPierre	R	S	R	R	K	R	Y	R	K	R	S
Panama KX573995.1 PA410	.	.	.	.	.	.	.	.	T	.	.
Panama KX573992.1 PA385	.	.	.	.	.	.	.	.	T	.	.
Panama KX573985.1 PA333	.	.	.	.	.	.	.	.	T	.	.
Panama KX573954.1 PA091	.	.	.	.	.	.	.	.	T	.	.
Panama KX573951.1 PA084	.	.	.	.	.	.	.	.	T	.	.
Panama KX573948.1 PA072	.	.	.	.	.	.	.	.	T	.	.
Panama KX573977.1 PA206	.	.	.	G	T	A	.	.	T	A	C
