# Supplementary material for: Genome Analysis of Triploid Hybrid Leishmania Parasite from the Neotropics
Source: Emerg Infect Dis. 2023 May;29(5):1076–8. doi: 10.3201/eid2905.221456 (PMC10124652; doi:10.3201/eid2905.221456)
Supplement: Appendix 2 — Additional whole genome information about genome analysis of triploid hybrid Leishmania parasite from the Neotropics. [file 22-1456-Techapp-s2.pdf]

Article DOI: https://doi.org/10.3201/eid2905.221456

*EID cannot ensure accessibility for supplementary materials supplied by authors. Readers who have difficulty accessing supplementary content should contact the authors for assistance.*

# Genome Analysis of Triploid Hybrid *Leishmania* Parasite from Neotropics

## Appendix 2

### Genome sequence analyses of MHOM/CR/2020/StPierre

### PART A: Decomposing of haplotype sequences for the HSP70 gene

The ambiguity of bases at 11 positions in the HSP70 fragment (Table 1) prompted us to decompose haplotype sequences to infer the parental species of MHOM/CR/2020/StPierre. To this end, 150bp read sequences were mapped with SMALT against one of the HSP70 genes within the *L. braziliensis* M2904 reference genome. Sequence data spanning all six neighboring ambiguous positions (1576–1679) in one read were extracted. This revealed five sets of identical sequences (i.e., haplotypes) found within a total of 83 high-quality read sequences. Haplotype 1 (H1) was found in 26 read sequences (32%) and has alleles equal to the *L. braziliensis* M2904 reference (Table 1). Haplotype 2 (H2) was found in 31 read sequences (38%) and contains alleles specific to *L. guyanensis* (Table 1). The remaining 3 haplotypes (26 sequences) showed signatures of mosaic ancestry, with allelic combinations similar to haplotypes 1 and 2.

### PART B: Whole genome sequence analyses

#### Publicly available sequence data

To capture as much as possible the genome diversity of *Leishmania (Viannia)* in South-America, we included publicly available sequence data from 35 strains and generated sequence data for 5 strains. Specifically, we included sequence data of the following seven *Leishmania (Viannia)* species: *L. lainsoni* (N = 1) (*1*), *L. naiffi* (N = 3) (*2–4*), *L. shawi* (N = 1) (*4*), *L.*

*guyanensis* (N = 3) (*2–5*), *L. panamensis* (N = 15) (*4*,*6–8*), *L. peruviana* (N = 2) (*7*) and *L. braziliensis* (N = 15) (*7*,*9*) (Table 2).

The three *L. guyanensis* strains originated from French Guiana, Brazil and Venezuela. The 15 *L. panamensis* strains originated from Colombia (N = 13) and Panama (N = 2) (Table 2). Note that one strain (LgCL085) was previously considered as *L. guyanensis* (*3*), but was here classified as *L. panamensis* following the phylogenomic analyses presented below.

We considered a total of four genetically divergent subgroups within the *L. braziliensis* species complex. The first subgroup, here-after referred to as *L. braziliensis* 1, is regarded as the main *L. braziliensis* species and is responsible for human (muco-)cutaneous leishmaniasis. The second subgroup, here-after referred to as *L. braziliensis* 2, has been occasionally diagnosed in Peru and Bolivia (*10–12*). The third subgroup, here-after referred to as *L. braziliensis* 3, was described within a geographically restricted ecotype in the Pernambuco state in Brazil (*13*). The fourth subgroup, here-after referred to as *L. braziliensis* 4, includes two strains from Colombia (*9*), and was classified as such by us following the phylogenomic analyses presented below.

**Genome sequencing of five *L. braziliensis* strains**

Sequence data was generated for three *L. braziliensis* 2 strains (CUM555, CUM663 and PER163) and two *L. braziliensis* 3 strains (HBO and LIS) (Table 2). To this end, parasites were grown in culture medium for 3 to 4 days at the Antwerp Institute of Tropical Medicine or FIOCRUZ in Brazil, and their DNA was extracted using a commercial column DNA extraction protocol. At the Wellcome Sanger Institute, genomic DNA was sheared into 400 to 600 bp fragments by focused ultrasonication (Covaris Inc.), and amplification-free Illumina libraries were prepared (*14*). One hundred base pair paired end reads were generated on the HiSeq 2000, and 150 bp paired end reads were generated on the HiSeq ×10 according to the manufacturer's standard sequencing protocol.

**Bioinformatic analyses**

Genomic sequence reads of all strains were mapped against the *L. braziliensis* M2904 reference genome (available on https://tritrypdb.org as LbraziliensisMHOMBR75M2904_2019) using SMALT v0.7.6 (https://www.sanger.ac.uk/tool/smalt-0/). This reference includes 35 major chromosomes (32.73Mb) and a complete mitochondrial maxicircle (27.69kb) (*7*).

Given the diversity of species included in this study, we tested the quality of sequence alignments against the M2904 reference by characterizing the accessible genome (*15*), i.e., genomic regions with a minimum read depth of 5, mapping quality of 25 and base quality of 25. The coverage of the accessible genome ranged between 28.6Mb (87.2% of the chromosomal region) for *L. shawi* strain M8408 and 30.4Mb (92.9%) for *L. braziliensis* strain Lb8025 (Table 3). The coverage for strain MHOM/CR/2020/StPierre was equal to 30.0 Mb (91.8%) (Table 3). These results showed that a substantial fraction of the reference genome can be used for genotyping and ancestry analyses.

**Large number of heterozygous sites (where one haplotype is similar to M2904) points to a member of the *L. braziliensis* species complex as one of the parents**

A total of 125,632 SNPs were identified within the strain from Costa Rica, including 21,168 homozygous SNPs (where both haplotypes were different to the consensus sequence of M2904) and 104,464 heterozygous SNPs (where one haplotype was similar to M2904 and the other different). This latter observation indicates *L. braziliensis* as one of the species that contributed sequences. This number of heterozygous SNPs was at least 4.1 and 16.4 times higher compared to the numbers observed for *L. braziliensis* and *L. guyanensis* respectively (Figure 1), confirming that the Costa Rica strain is either the result of hybridization or a mixed infection.

**Phylogenetic analyses based on the mitochondrial maxicircle points to a member of the *L. guyanensis* species complex as one of the parents**

Single Nucleotide Polymorphisms (SNPs) were called jointly across all genomes using bcftools mpileup/call (*16*), retaining only SNPs with a minimum SNP quality (QUAL) of 100, mapping quality of 50 and genotype quality of 60. Genotyping uncovered - across all genomes - a total 1,103,461 SNPs within the 35 major chromosomes and 467 SNPs within the coding region of the mitochondrial maxicircle. A phylogenetic network reconstructed with SplitsTree (*17*) using nuclear SNPs showed that the Costa Rica strain occupied a central position between the *L. guyanensis* and *L. braziliensis* species complexes, highlighting its uncertain ancestry (Figure 2). A phylogeny based on the uniparentally inherited mitochondrial maxicircles (*7*,*18*,*19*) showed that the Costa Rica strain clustered with *L. guyanensis* (Figure 3), pointing to a member of the *L. guyanensis* species complex as one of the parental species.

**Genomic distribution of heterozygous sites suggests that MHOM/CR/2020/StPierre is a hybrid parasite, rather than the result of a mixed infection**

When analyzing the genome-wide SNP distribution in non-overlapping 10kb windows (3,091 windows in total), 2,890 windows (93.5%) were found where at least half the SNPs were heterozygous (Figure 4), and only 108 windows (3.5%) were entirely homozygous. The majority of homozygous 10kb windows (95/108, 88%) covered almost entirely chromosomes 1 and 11, the first 140 kb of chromosome 20 and the last 60kb of chromosome 27 (Figure 5). The observation of a largely heterozygous genome that is interrupted by homozygous stretches strongly suggests that the isolate is a hybrid parasite, rather than the result of a mixed infection.

**Distribution of allelic read depths at heterozygous sites shows that the hybrid is a triploid parasite, and reveals major recombination breakpoints**

The genetic complexity of *Leishmania* infections is of particular interest because of the existence of aneuploidy, which was genomically inferred by investigating allelic read depth frequencies (ARDF) at heterozygous sites and standardized chromosomal read depths (*20*). The genome-wide ARDF distribution was bimodal for the hybrid parasite, with modes 0.33 and 0.67 (Figure 6) suggesting that the hybrid is triploid (*21*), with the exceptions of chromosomes 1 and 11 (no distribution because of absence of heterozygous sites), 3 and 12 (trimodal distributions). Assuming triploidy, standardized chromosomal read depths showed that chromosome 10 was trisomic, chromosomes 1, 3 and 12 were tetrasomic and chromosome 31 was hexasomic (Table 4). Shifts in the ARDF distribution of species-specific alleles between the two modes along chromosomes (Figure 7) represent recombination events that occurred since the hybridization event (*18*) and further exclude the possibility of a mixed infection.

**Neighbor-Joining phylogenetic trees**

The low bootstrap estimates in the ML phylogenetic trees based on SNPs detected in the telomeric region of chromosome 20 (Figure in main article) and the maxicircle coding region (Figure 3) prompted us to reconstruct a different type of phylogeny for complementary insights. To this end, we reconstructed Neighbor-Joining phylogenetic trees based on the number of nucleotide differences between *Leishmania* strains using the R package ape (*23*), which are shown in Figures 8 and 9.

## References


1. Lin W, Batra D, Narayanan V, Rowe LA, Sheth M, Zheng Y, et al. First draft genome sequence of Leishmania (Viannia) lainsoni strain 216-34, isolated from a Peruvian clinical case. Microbiol Resour Announc. 2019;8:e01524–18. PubMed https://doi.org/10.1128/MRA.01524-18

2. Patiño LH, Muñoz M, Pavia P, Muskus C, Shaban M, Paniz-Mondolfi A, et al. Filling the gaps in Leishmania naiffi and Leishmania guyanensis genome plasticity. G3 (Bethesda). 2022;12:jkab377. PubMed https://doi.org/10.1093/g3journal/jkab377

3. Coughlan S, Taylor AS, Feane E, Sanders M, Schonian G, Cotton JA, et al. *Leishmania naiffi* and *Leishmania guyanensis* reference genomes highlight genome structure and gene evolution in the *Viannia* subgenus. R Soc Open Sci. 2018;5:172212. PubMed https://doi.org/10.1098/rsos.172212

4. Harkins KM, Schwartz RS, Cartwright RA, Stone AC. Phylogenomic reconstruction supports supercontinent origins for *Leishmania*. Infect Genet Evol. 2016;38:101–9. PubMed https://doi.org/10.1016/j.meegid.2015.11.030

5. Batra D, Lin W, Rowe LA, Sheth M, Zheng Y, Loparev V, et al. Draft genome sequence of French Guiana *Leishmania* (*Viannia*) *guyanensis* strain 204-365, assembled using long reads. Microbiol Resour Announc. 2018;7:e01421-18. PubMed https://doi.org/10.1128/MRA.01421-18

6. Llanes A, Restrepo CM, Del Vecchio G, Anguizola FJ, Lleonart R. The genome of *Leishmania panamensis*: insights into genomics of the L. (Viannia) subgenus. Sci Rep. 2015;5:8550. PubMed https://doi.org/10.1038/srep08550

7. Van den Broeck F, Savill NJ, Imamura H, Sanders M, Maes I, Cooper S, et al. Ecological divergence and hybridization of Neotropical *Leishmania* parasites. Proc Natl Acad Sci U S A. 2020;117:25159–68. PubMed https://doi.org/10.1073/pnas.1920136117

8. Patino LH, Muñoz M, Muskus C, Méndez C, Ramírez JD. Intraspecific genomic divergence and minor structural variations in *Leishmania (Viannia) panamensis.* Genes (Basel). 2020;11:252. PubMed https://doi.org/10.3390/genes11030252

9. Patino LH, Muñoz M, Cruz-Saavedra L, Muskus C, Ramírez JD. Genomic diversification, structural plasticity, and hybridization in *Leishmania (Viannia) braziliensis.* Front Cell Infect Microbiol. 2020;10:582192. PubMed https://doi.org/10.3389/fcimb.2020.582192

10. Odiwuor S, Veland N, Maes I, Arévalo J, Dujardin JC, Van der Auwera G. Evolution of the *Leishmania braziliensis* species complex from amplified fragment length polymorphisms, and clinical implications. Infect Genet Evol. 2012;12:1994–2002. PubMed https://doi.org/10.1016/j.meegid.2012.03.028

11. Brilhante AF, Lima L, Zampieri RA, Nunes VLB, Dorval MEC, Malavazi PFNDS, et al. Leishmania (Viannia) braziliensis type 2 as probable etiological agent of canine cutaneous leishmaniasis in Brazilian Amazon. PLoS One. 2019;14:e0216291. PubMed https://doi.org/10.1371/journal.pone.0216291

12. Van der Auwera G, Ravel C, Verweij JJ, Bart A, Schönian G, Felger I. Evaluation of four single-locus markers for Leishmania species discrimination by sequencing. J Clin Microbiol. 2014;52:1098–104. PubMed https://doi.org/10.1128/JCM.02936-13

13. S L Figueiredo de Sá B, Rezende AM, Melo Neto OP, Brito MEF, Brandão Filho SP. Identification of divergent Leishmania (Viannia) braziliensis ecotypes derived from a geographically restricted area through whole genome analysis. PLoS Negl Trop Dis. 2019;13:e0007382. PubMed https://doi.org/10.1371/journal.pntd.0007382

14. Kozarewa, Ning, Quail, Sanders. Amplification-free Illumina sequencing-library preparation facilitates improved mapping and assembly of (G+ C)-biased genomes. Nat Methods. 2009;6:291–5. **PMID 19287394**

15. McKenna A, Hanna M, Banks E, Sivachenko A, Cibulskis K, Kernytsky A, et al. The genome analysis toolkit: a MapReduce framework for analyzing next-generation DNA sequencing data. Genome Res. 2010;20:1297–303. PubMed https://doi.org/10.1101/gr.107524.110

16. Danecek P, Bonfield JK, Liddle J, Marshall J, Ohan V, Pollard MO, et al. Twelve years of SAMtools and BCFtools. Gigascience. 2021;10:giab008. PubMed https://doi.org/10.1093/gigascience/giab008

17. Huson DH. SplitsTree: analyzing and visualizing evolutionary data. Bioinformatics. 1998;14:68–73. PubMed https://doi.org/10.1093/bioinformatics/14.1.68

18. Rogers MB, Downing T, Smith BA, Imamura H, Sanders M, Svobodova M, et al. Genomic confirmation of hybridisation and recent inbreeding in a vector-isolated Leishmania population. PLoS Genet. 2014;10:e1004092. PubMed https://doi.org/10.1371/journal.pgen.1004092

19. Van den Broeck F, Tavernier LJM, Vermeiren L, Dujardin JC, Van Den Abbeele J. Mitonuclear genomics challenges the theory of clonality in Trypanosoma congolense: reply to Tibayrenc and Ayala. Mol Ecol. 2018;27:3425–31. PubMed https://doi.org/10.1111/mec.14809

20. Rogers MB, Hilley JD, Dickens NJ, Wilkes J, Bates PA, Depledge DP, et al. Chromosome and gene copy number variation allow major structural change between species and strains of Leishmania. Genome Res. 2011;21:2129–42. PubMed https://doi.org/10.1101/gr.122945.111

21. Tihon E, Imamura H, Dujardin JC, Van Den Abbeele J, Van den Broeck F. Discovery and genomic analyses of hybridization between divergent lineages of Trypanosoma congolense, causative agent of Animal African Trypanosomiasis. Mol Ecol. 2017;26:6524–38. PubMed https://doi.org/10.1111/mec.14271

22. Leys S, Windsor-Reid P. Phylogenetic Tree for Porifera Wnts using IQTREE. 2017 [cited 2019 Aug 15]. https://era.library.ualberta.ca/items/ca816413-df15-4a31-8da8-3b06077387b9

23. Paradis E, Schliep K. ape 5.0: an environment for modern phylogenetics and evolutionary analyses in R. Bioinformatics. 2019;35:526–8. PubMed https://doi.org/10.1093/bioinformatics/bty633

**Table 1.** Sequence variation across 11 positions in the multicopy heat-shock protein 70 gene (*hsp70*)*

| POSITION on full CDS | SANGER | WGS | H1 (M2904) | H2 (*L. guyanensis*) |
|---|---|---|---|---|
| 504 | R | G, A | NA | NA |
| 731 | S | G, C | NA | NA |
| 762 | R | G, A | NA | NA |
| 1173 | R | G, A | NA | NA |
| 1576 | K | T, G | T | G |
| 1600 | R | A, G | A | G |
| 1632 | Y | C, T | C | T |
| 1666 | R | G, A | G | A |
| 1669 | K | T, G | T | G |
| 1679 | R | A, G | A | G |
| 1734 | S | C, G | NA | NA |

*A 1,245 bp fragment of the *hsp70* locus was sequenced for species typing, revealing ambiguous bases at 11 positions (column 'SANGER'). Underlying alleles were identified through genotyping of Single Nucleotide Polymorphisms (SNPs) after mapping sequencing reads against a single *hsp70* gene sequence of the M2904 reference genome. This revealed heterozygous SNPs at all 11 ambiguous positions: the two alleles in column 'WGS' represent the reference allele (similar to the M2904 consensus sequence) and alternate allele (different to the M2904 consensus sequence), respectively. Haplotypes (columns 'H1' and 'H2') were decomposed for six neighboring ambiguous positions (1576–1679) by extracting 150bp sequence reads covering all six positions.

**Table 2.** List of 40 publicly and in-house available sequence data from seven *Leishmania (Viannia)* species that were used for whole genome sequence analyses*

| SRA Run Accession Number | Name | Species | Country | References |
|---|---|---|---|---|
| ERR471302 | CUM29 | *L. braziliensis* 1 | Bolivia | (*7*) |
| ERR3988461 | Lb7529 | *L. braziliensis 1* | Colombia | (*9*) |
| ERR3988463 | Lb7740 | *L. braziliensis 1* | Colombia | (*9*) |
| ERR3988465 | Lb7933 | *L. braziliensis 1* | Bolivia | (*9*) |
| ERR3988466 | Lb8025 | *L. braziliensis 1* | Colombia | (*9*) |
| ERR3988467 | Lb8102 | *L. braziliensis 1* | Colombia | (*9*) |
| ERR377654 | LC1565 | *L. braziliensis 1* | Peru | (*7*) |
| ERR3150801 | RO393 | *L. braziliensis 1* | Brazil | (*7*) |
| ERR877281 | CUM555 | *L. braziliensis 2* | Bolivia | this study |
| ERR877282 | CUM663 | *L. braziliensis 2* | Bolivia | this study |
| ERR467298 | PER163 | *L. braziliensis 2* | Peru | this study |
| ERR3150831, ERR3150735 | HBO | *L. braziliensis 3* | Brazil | this study |
| ERR3150728, ERR3150824 | LIS | *L. braziliensis 3* | Brazil | this study |
| ERR3988462 | Lb7616 | *L. braziliensis 4* | Colombia | (*9*) |
| ERR3988464 | Lb7864 | *L. braziliensis 4* | Colombia | (*9*) |
| SRR8179913 | 204–365 | *L. guyanensis* | French Guyana | (*5*) |
| SRR1662195 | M4147 | *L. guyanensis* | Brazil | (*4*) |
| ERR6188759 | S8104 | *L. guyanensis* | Venezuela | (*2*) |
| SRR8179821 | 216_34 | *L. lainsoni* | Peru | (*1*) |
| ERR6188758 | HOMI-81 | *L. naiffi* | Colombia | (*2*) |
| ERR205764 | LnCL223 | *L. naiffi* | Colombia | (*3*) |
| SRR1657911 | M5533 | *L. naiffi* | Brazil | (*4*) |
| ERR205773 | LgCL085 | *L. panamensis* | Colombia | (*3*) |
| ERR3648455 | LpS7762 | *L. panamensis* | Colombia | (*8*) |
| ERR3648456 | LpS7842 | *L. panamensis* | Colombia | (*8*) |
| ERR3648457 | LpS8036 | *L. panamensis* | Colombia | (*8*) |
| ERR3648458 | LpS8046 | *L. panamensis* | Colombia | (*8*) |
| ERR3648459 | LpS8049 | *L. panamensis* | Colombia | (*8*) |
| ERR3648460 | LpS8056 | *L. panamensis* | Colombia | (*8*) |
| ERR3648461 | LpS8061 | *L. panamensis* | Colombia | (*8*) |
| ERR3648463 | LpS8087 | *L. panamensis* | Colombia | (*8*) |
| ERR3648466 | LpS8117 | *L. panamensis* | Colombia | (*8*) |
| ERR3648467 | LpS8124 | *L. panamensis* | Colombia | (*8*) |
| ERR3648469 | LpS8136 | *L. panamensis* | Colombia | (*8*) |
| SRR1552486 | PSC1 | *L. panamensis* | Panama | (*6*) |
| ERR3656054 | REST417 | *L. panamensis* | Colombia | (*7*) |
| SRR1662198 | WR120 | *L. panamensis* | Panama | (*4*) |
| ERR662608 | HB83 | *L. peruviana* | Peru | (*7*) |
| ERR662626 | LCA04 | *L. peruviana* | Peru | (*7*) |
| SRR1657909 | M8408 | *L. shawi* | Brazil | (*4*) |

*RO393, LIS and HBO are *Leishmania* strains available at the *Leishmania* collection from the Oswaldo Cruz Foundation, Rio de Janeiro, Brazil (http://clioc.fiocruz.br/).

**Table 3.** Coverage and number of accessible genomic regions in each of the 41 *Leishmania* strains

| Species | Strain | Coverage | Fraction | Number of regions |
|---|---|---|---|---|
| *Leishmania shawi* | M8408 | 28552102 | 87,23% | 65585 |
| *Leishmania panamensis* | WR120 | 28586898 | 87,34% | 64401 |
| *Leishmania naiffi* | M5533 | 28604295 | 87,39% | 61373 |
| *Leishmania panamensis* | REST417 | 28765477 | 87,88% | 77484 |
| *Leishmania lainsoni* | 216_34 | 28788593 | 87,96% | 94726 |
| *Leishmania naiffi* | LnCL223 | 28832216 | 88,09% | 94858 |
| *Leishmania guyanensis* | M4147 | 28867457 | 88,20% | 50537 |
| *Leishmania naiffi* | HOM81 | 29366435 | 89,72% | 38873 |
| *Leishmania panamensis* | LgCL085 | 29410263 | 89,85% | 41655 |
| *Leishmania panamensis* | PSC1 | 29501473 | 90,13% | 30825 |
| *Leishmania guyanensis* | 204_365 | 29585997 | 90,39% | 62565 |
| *Leishmania panamensis* | LpS8124 | 29638319 | 90,55% | 24813 |
| *Leishmania panamensis* | LpS8136 | 29642314 | 90,56% | 25099 |
| *Leishmania panamensis* | LpS8117 | 29646217 | 90,58% | 23988 |
| *Leishmania panamensis* | LpS8049 | 29661360 | 90,62% | 23912 |
| *Leishmania panamensis* | LpS8061 | 29678344 | 90,67% | 24194 |
| *Leishmania panamensis* | LpS8046 | 29682946 | 90,69% | 24614 |
| *Leishmania guyanensis* | S8104 | 29684216 | 90,69% | 23465 |

| Species | Strain | Coverage | Fraction | Number of regions |
|---|---|---|---|---|
| *Leishmania panamensis* | LpS8056 | 29686912 | 90,70% | 24218 |
| *Leishmania panamensis* | LpS7762 | 29692655 | 90,72% | 21681 |
| *Leishmania panamensis* | LpS7842 | 29715153 | 90,79% | 22423 |
| *Leishmania panamensis* | LpS8087 | 29716249 | 90,79% | 21868 |
| *Leishmania panamensis* | LpS8036 | 29728357 | 90,83% | 21984 |
| *Leishmania braziliensis 2* | PER163 | 29791821 | 91,02% | 32749 |
| *Leishmania braziliensis 4* | Lb7864 | 29815765 | 91,09% | 17887 |
| *Leishmania braziliensis 1* | LC1565 | 29861013 | 91,23% | 27927 |
| *Leishmania braziliensis 2* | CUM663 | 29872352 | 91,27% | 32131 |
| *Leishmania braziliensis 2* | CUM555 | 29887936 | 91,31% | 31159 |
| *Leishmania braziliensis 4* | Lb7616 | 29944008 | 91,49% | 15486 |
| *Leishmania peruviana* | HB83 | 29984415 | 91,61% | 29030 |
| *Leishmania peruviana* | LCA04 | 30014816 | 91,70% | 25532 |
| *Leishmania braziliensis 1* | CUM29 | 30025178 | 91,73% | 21921 |
| *Hybrid strain* | MHOM/CR/2020/StPierre | 30032613 | 91,76% | 16291 |
| *Leishmania braziliensis 3* | HBOA1 | 30039532 | 91,78% | 28782 |
| *Leishmania braziliensis 3* | LISA1 | 30046468 | 91,80% | 27611 |
| *Leishmania braziliensis 1* | Lb8102 | 30054846 | 91,82% | 11135 |
| *Leishmania braziliensis 1* | Lb7740 | 30062478 | 91,85% | 11505 |
| *Leishmania braziliensis 1* | Lb7529 | 30082397 | 91,91% | 10581 |
| *Leishmania braziliensis 1* | Lb7933 | 30129942 | 92,05% | 10957 |
| *Leishmania braziliensis 1* | RO393 | 30160429 | 92,15% | 27477 |
| *Leishmania braziliensis 1* | Lb8025 | 30412185 | 92,92% | 8012 |

**Table 4.** Somy variation was genomically inferred by investigating allelic read depth frequencies (ARDF) at heterozygous sites and standardized chromosomal read depths assuming triploidy (*20*)

| Chromosome | Haploid Chromosomal Read Depths | Somy based on read depths, assuming triploidy | Somy based on ARDF |
|---|---|---|---|
| 1 | 1,4 | 4,3 | NA |
| 2 | 0,9 | 2,6 | 3 |
| 3 | 1,3 | 3,9 | 4 |
| 4 | 1,0 | 2,9 | 3 |
| 5 | 1,0 | 3,1 | 3 |
| 6 | 1,0 | 3,0 | 3 |
| 7 | 1,0 | 3,0 | 3 |
| 8 | 1,0 | 2,9 | 3 |
| 9 | 1,0 | 3,0 | 3 |
| 10 | 0,9 | 2,8 | 3 |
| 11 | 1,1 | 3,2 | NA |
| 12 | 1,4 | 4,1 | 4 |
| 13 | 1,0 | 2,9 | 3 |
| 14 | 1,0 | 3,0 | 3 |
| 15 | 1,0 | 3,1 | 3 |
| 16 | 1,0 | 3,0 | 3 |
| 17 | 1,0 | 3,1 | 3 |
| 18 | 1,0 | 3,0 | 3 |
| 19 | 1,0 | 2,9 | 3 |
| 20 | 1,0 | 2,9 | 3 |
| 21 | 1,0 | 3,0 | 3 |
| 22 | 1,0 | 3,0 | 3 |
| 23 | 1,0 | 3,1 | 3 |
| 24 | 1,0 | 3,0 | 3 |
| 25 | 1,0 | 3,1 | 3 |
| 26 | 1,0 | 3,0 | 3 |
| 27 | 1,0 | 3,0 | 3 |
| 28 | 1,0 | 3,0 | 3 |
| 29 | 1,0 | 3,0 | 3 |
| 30 | 1,0 | 3,0 | 3 |
| 31 | 2,0 | 6,0 | 6 |
| 32 | 1,0 | 3,0 | 3 |
| 33 | 1,0 | 3,0 | 3 |
| 34 | 1,0 | 3,0 | 3 |
| 35 | 1,0 | 3,0 | 3 |

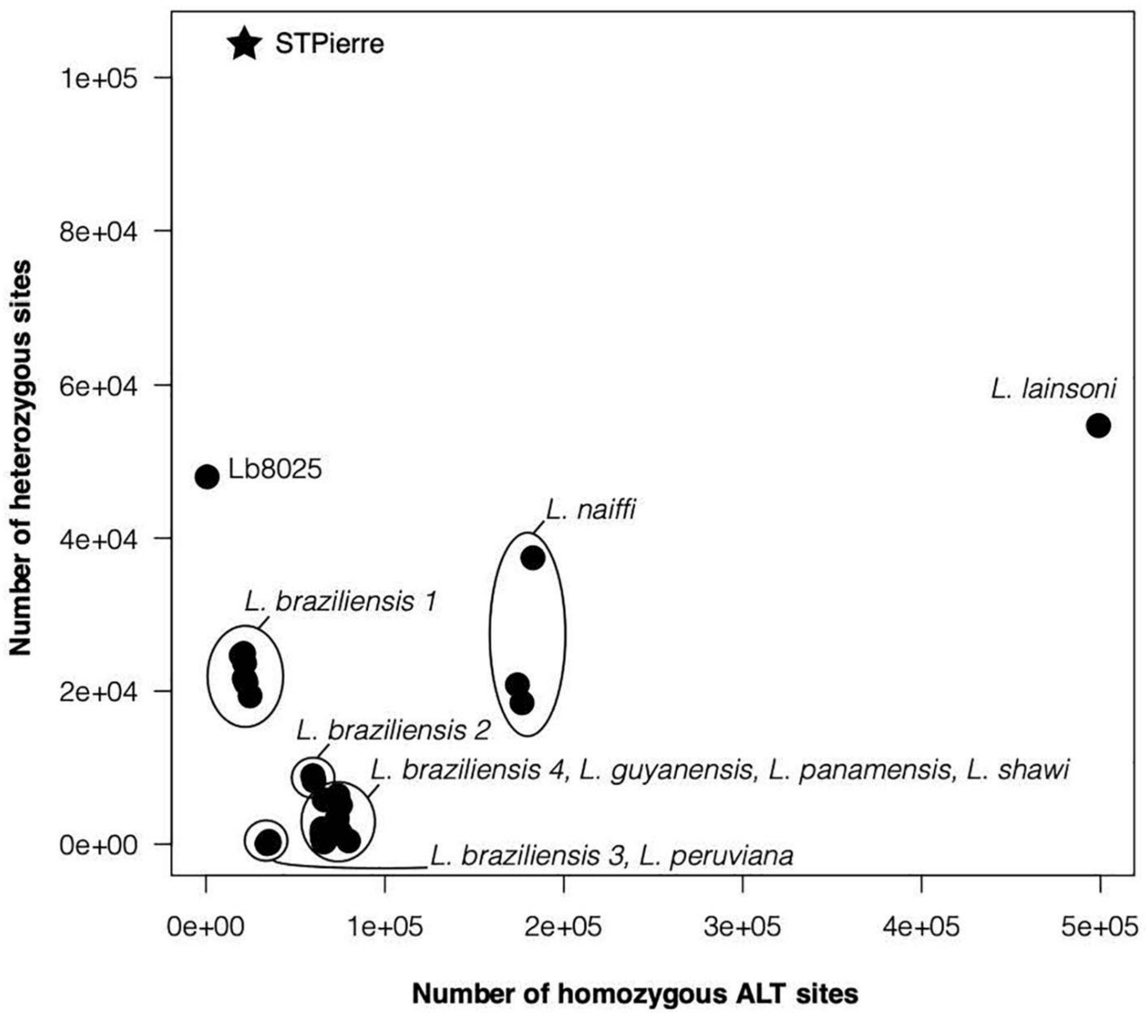


**Appendix 2 Figure 1.** Number of heterozygous versus homozygous sites for each of the 41 *Leishmania* genomes included in this study. ALT = alternate alleles, i.e., alleles different to the reference genome.

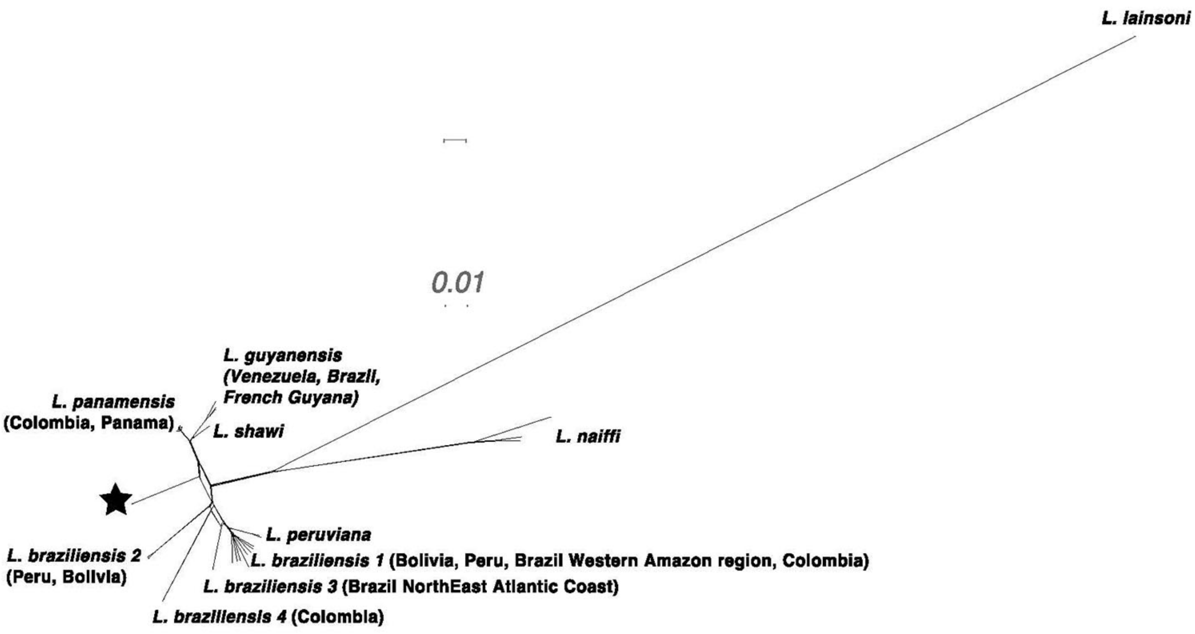


**Appendix 2 Figure 2.** Phylogenetic network as obtained with SplitsTree (*17*) using 1,103,461 genome-wide SNPs called across 41 *Leishmania* genomes. The star indicates the position of MHOM/CR/2020/StPierre. For each strain, sequences were composed based on concatenated SNPs that were each coded by two base pairs, resulting in sequences of 2,206,922 nt. The distance scale on top shows substitutions/sites.

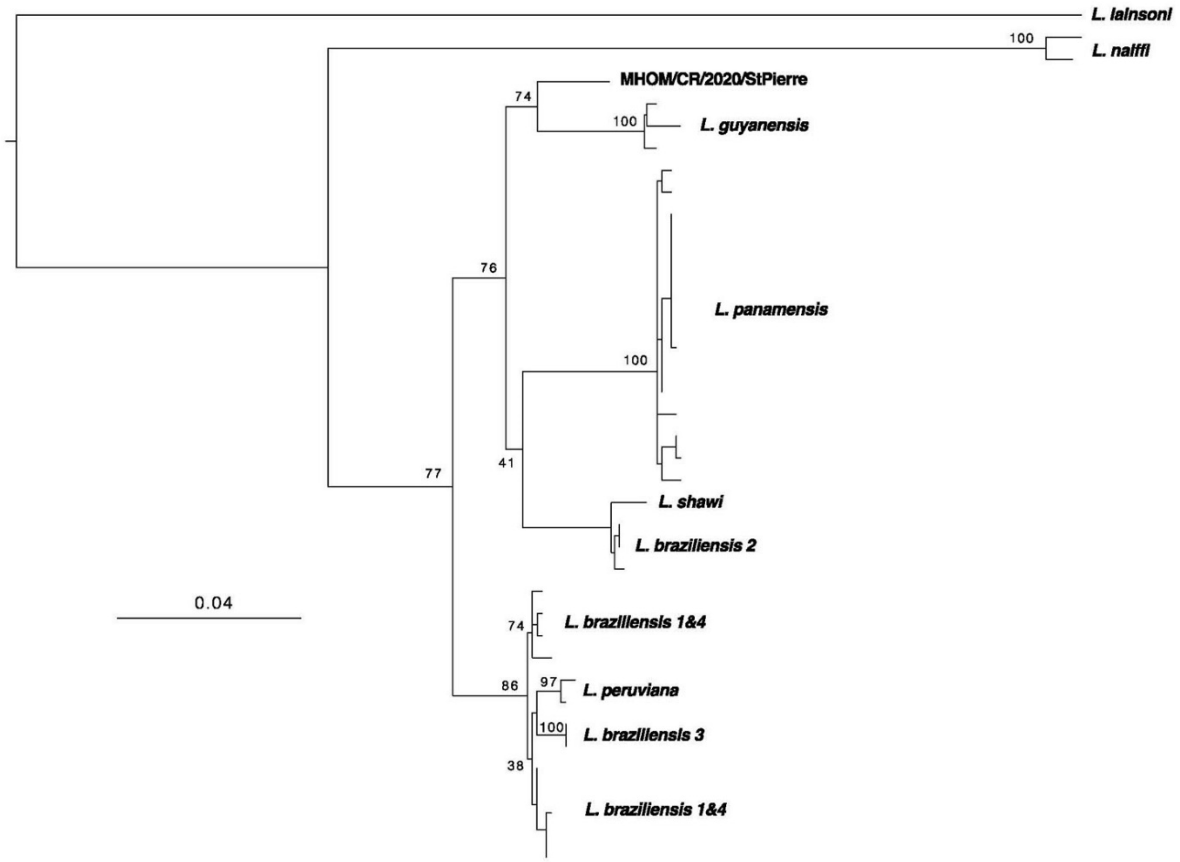


**Appendix 2 Figure 3.** Midpoint rooted Maximum Likelihood phylogenetic trees based on 467 SNPs called within the mitochondrial maxicircle coding region. Consensus phylogenetic trees were generated from 1,000 bootstrap trees using IQTREE (*22*) under the TN+F+ASC substitution model (TN = unequal transition/transversion rates and unequal purine/pyrimidine rates, *F* = empirical base frequencies, ASC = ascertainment bias correction), which was the best-fit model revealed by ModelFinder as implemented in IQTREE. Assuming that the maxicircle is haploid and because we observed no signatures of heteroplasmy (all SNPs were homozygous), SNPs were coded by one base pair, resulting in sequences of 467 nt. The distance scale (bottom) shows substitutions/sites. Branch support values are presented near each node following 1000 bootstrap replicates. Note that the *L. braziliensis* lineage 2 is closely related to *L. shawi* and positioned with low bootstrap support (41%) within the *L. guyanensis* species complex. This discrepancy between the nuclear (Figure 2) and maxicircle (Figure 3) phylogenies suggest a complex ancestry for the *L. braziliensis* lineage 2, an observation that warrants more detailed analyses in future research.

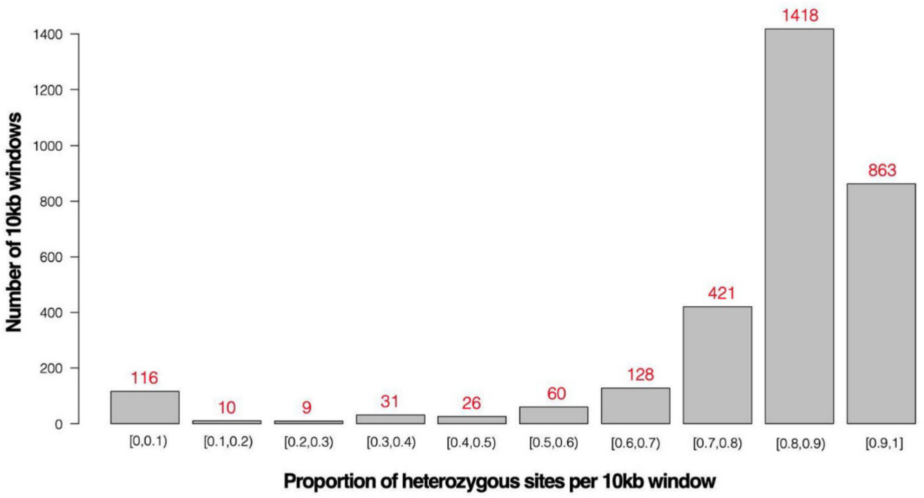


**Appendix 2 Figure 4.** Number of 10kb windows (y-axis) with a given fraction of heterozygous SNP sites (x-axis).

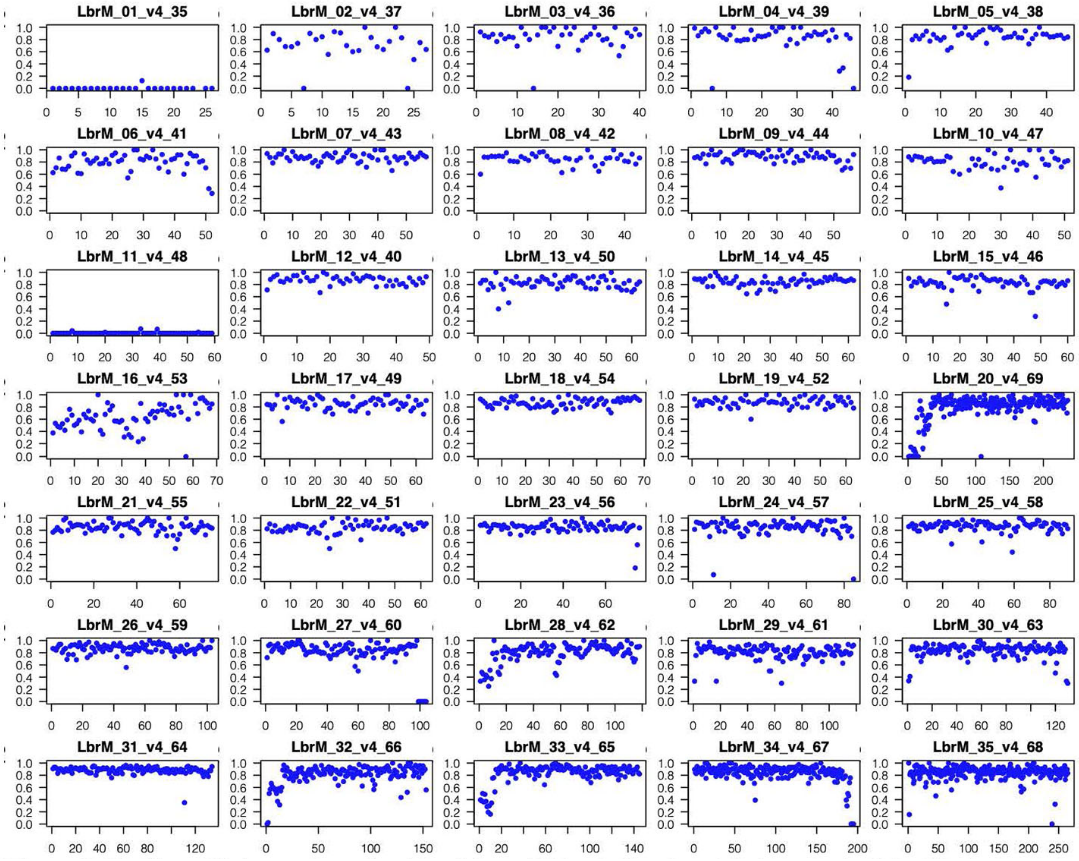


**Appendix 2 Figure 5.** Fraction of heterozygous sites (y-axis) per 10kb window (x-axis) along each of the 35 chromosomes. The majority of homozygous 10kb windows (95/108, 88%) covered almost entirely chromosomes 1 and 11, the first 140 kb of chromosome 20 and the last 60kb of chromosome 27.

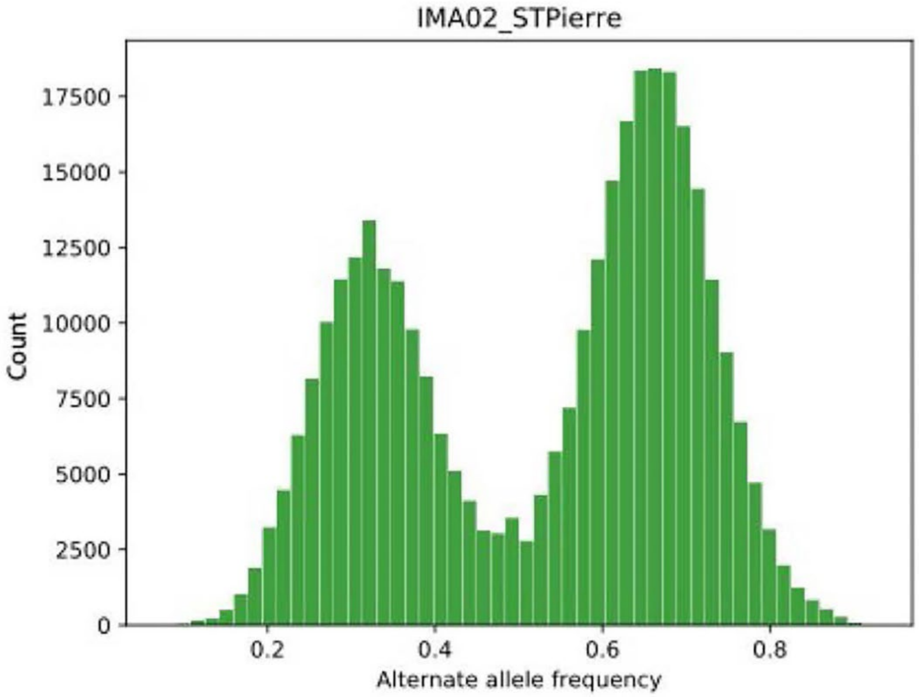


**Appendix 2 Figure 6.** Genome-wide distribution of read depth frequencies of alternate alleles at heterozygous sites for MHOM/CR/2020/StPierre.

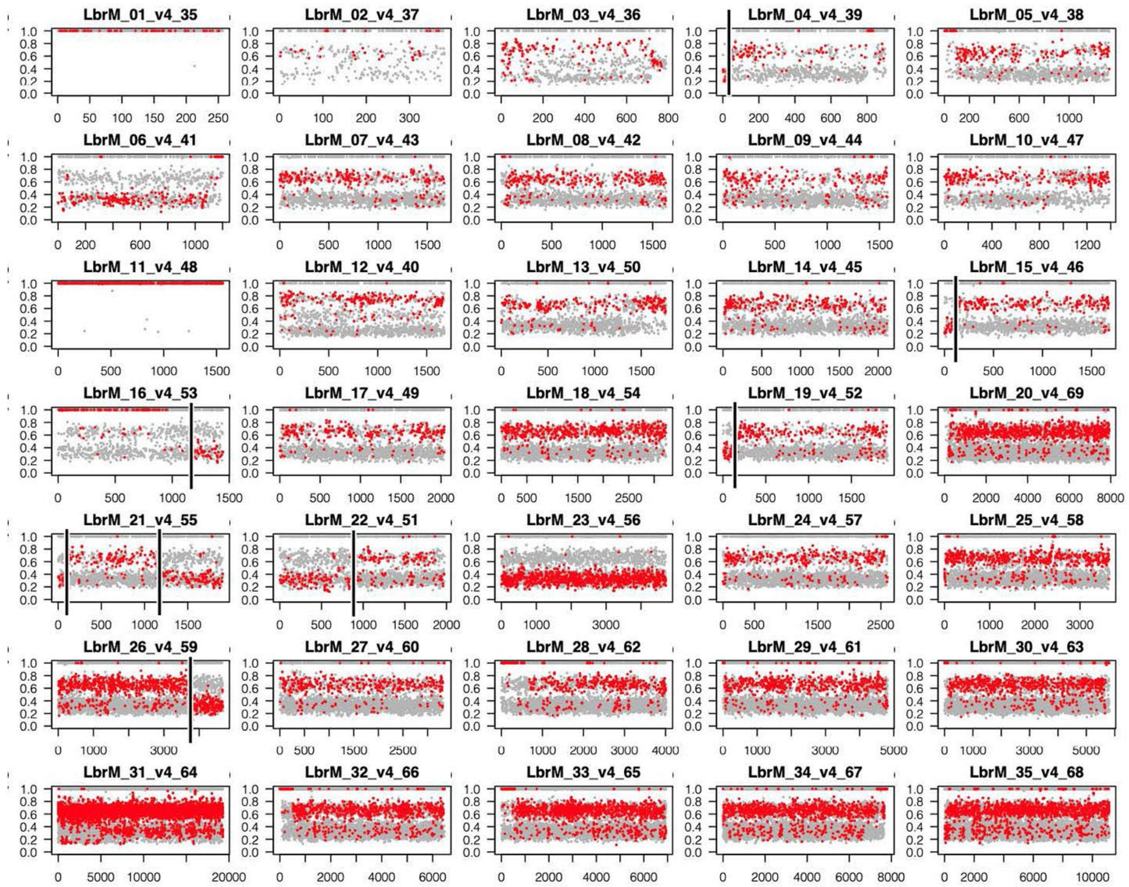


**Appendix 2 Figure 7.** Distribution of read depth frequencies of all alternate alleles (gray and red dots) at heterozygous sites for each of the 35 chromosomes in the hybrid genome. Red dots reflect alternate alleles that were specific to the *L. guyanensis* species complex, i.e., alleles found in *L. guyanensis* and/or *L. panamensis* and/or *L. shawi* strains and not in any other *Viannia* species. Black bars denote position of major shifts in read depth frequencies, which represent recombination events since the initial hybridization event.

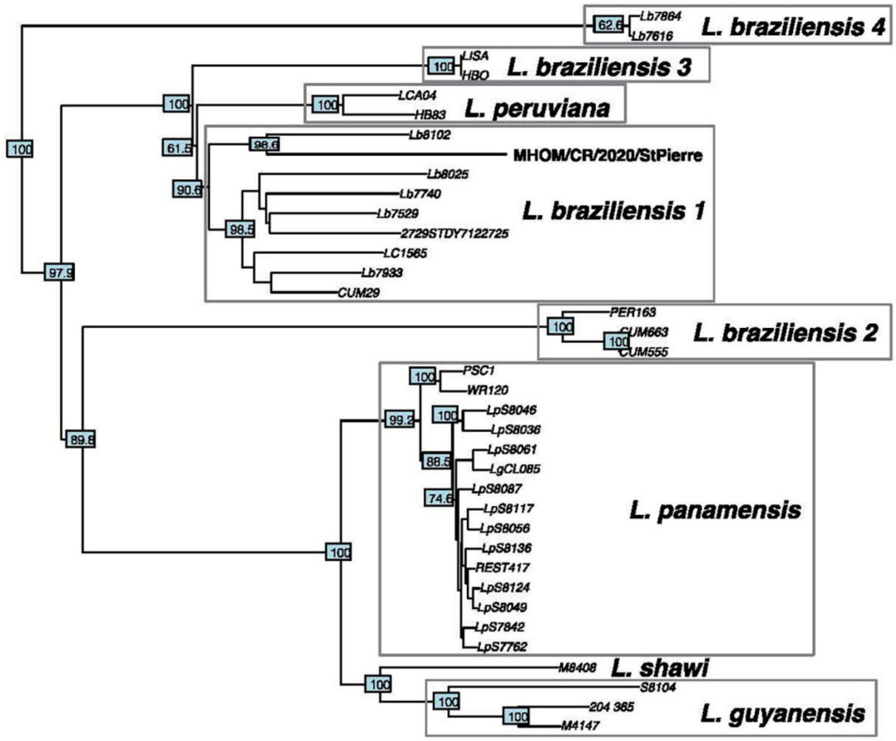


**Appendix 2 Figure 8.** Neighbor-Joining tree based on the number of nucleotide differences between *Leishmania* strains. Sequences contained 3,015 nt from the telomeric region of chromosome 20 and are the same as used in Figure 1 of the main text. Node support is based on 1,000 bootstrapped phylogenetic trees.

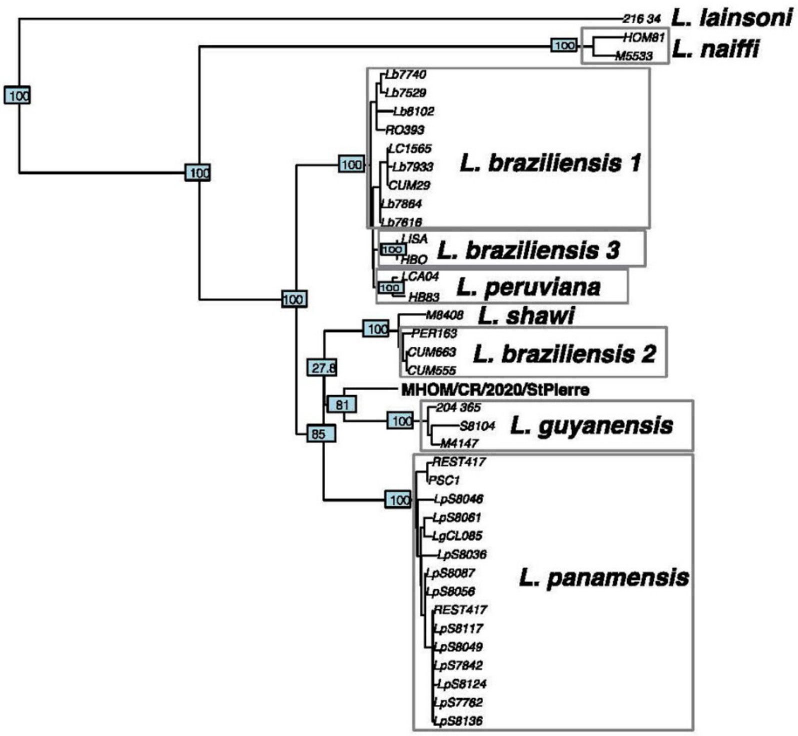


**Appendix 2 Figure 9.** Neighbor-Joining tree based on the number of nucleotide differences between *Leishmania* strains. Sequences contained 467 nt from the maxicircle coding region and are the same as used in Figure 3 of Appendix 2. Node support is based on 1000 bootstrapped phylogenetic trees.
